# Effects of Weight Loss on FGF-21 in Human Subjects: An Exploratory Study

**DOI:** 10.3390/ijerph16234877

**Published:** 2019-12-03

**Authors:** Michelle L. Headland, Peter M. Clifton, Jennifer B. Keogh

**Affiliations:** 1 School of Pharmacy and Medical Sciences, University of South Australia, Frome Road, Adelaide, SA 5000, Australia; peter.clifton@unisa.edu.au (P.M.C.); Jennifer.keogh@unisa.edu.au (J.B.K.); 2 Alliance for Research in Exercise, Nutrition and Activity (ARENA), University of South Australia, Frome Road, Adelaide, SA 5000, Australia; 3 Sansom Institute for Health Research University of South Australia, Frome Road, Adelaide, SA 5000, Australia

**Keywords:** intermittent energy restriction, fibroblast growth factor-21, weight loss

## Abstract

Fibroblast growth factor-21 (FGF-21), is a protein involved in cell growth and differentiation, development, wound repair and metabolism. Research looking at the impact of weight loss on FGF-21 levels is limited. The objective of this exploratory study was to determine changes in serum FGF-21 levels following weight loss induced by either continuous energy restriction or intermittent energy restriction. A sub cohort of participants who completed a 12-month dietary intervention trial following continuous energy restriction, or a week-on week-off energy restriction pattern, were selected for analysis. FGF-21 levels were not altered by weight loss and were not correlated with body weight or BMI at baseline or 12 months. Weight loss after 12 months either through continuous energy restriction or intermittent energy restriction was −5.9 ± 4.5 and −4.9 ± 3.4 kg, respectively. There was no change in FGF-21 levels, 0.3 ± 0.9 and 0.04 ± 0.2 ng/mL (*p* = 0.2). In conclusion, weight loss in healthy overweight or obesity subjects did not affect FGF-21 levels.

## 1. Introduction

As obesity rates increase worldwide, so do the rates of associated cardiometabolic conditions, including cardiovascular disease, non-alcoholic liver disease and type 2 diabetes-mellitus. The levels of morbidity and mortality linked to these conditions highlight the importance of identifying novel metabolic regulators of energy balance. 

Fibroblast growth factor-21 (FGF-21), one such emerging regulator, is a protein involved in cell growth and differentiation, development, wound repair and metabolism [1]. It is predominately produced in the liver and promotes fatty acid oxidation, increased energy expenditure and improved insulin sensitivity [1,2]. Several studies have found FGF-21 levels are correlated with BMI, waist circumference and visceral adipose tissues, suggesting that obesity is an FGF-21 resistant state [3]. Recently FGF-21 has been shown to be induced in the liver during fasting [4,5,6], and has been linked to the adaptive response following periods of starvation with a range of effects being witnessed, including altering metabolism and blunting of the growth hormone signalling pathway [7].

Current research suggests that normal FGF-21 levels within human subjects vary greatly (21–5300 pg/mL) and the impact of weight loss produces mixed results [8,9,10,11,12]. With research assessing the impact of weight loss in humans on FGF-21 levels being limited, and none comparing the difference following weight loss on a continuous energy restriction (CER) pattern to weight loss following an intermittent energy restriction (IER) pattern, the aim of this exploratory study was to determine if serum FGF-21 levels changed following weight loss induced by either CER or IER.

## 2. Materials and Methods

### 2.1. Study Methods

A 12-month randomised controlled trial was conducted to determine the effect of IER vs. CER on weight loss and weight loss maintenance in healthy adults with overweight or obesity. Results from this study have been reported [13]. Individuals followed an energy restriction of 4200 kJ/day for women or 5500 kJ/day for men in either a continuous pattern or intermittent pattern. Blood samples were taken at baseline and 12 months for measurement of FGF-21. FGF-21 was analysed on a sub cohort (*n* = 43) of participants who completed the energy restriction. Participants visited the research centre eight times and received dietetic support at each of these visits.

### 2.2. Weight and Height

Participants’ height was measured (first visit only) whilst barefoot using a stadiometer (SECA, Hamburg, Germany). Measurements were recorded to the nearest 0.1 cm. Body weight was recorded to the nearest 0.05 kg at all eight visits using calibrated digital scales (SECA, Hamburg, Germany). Participants attended after an overnight fast of 12 h from 8 pm the night before, barefoot and in light clothing.

### 2.3. Laboratory Analysis

Fasting blood samples were collected from a brachial vein for measurement of serum FGF-21 levels at baseline, following 8 weeks of energy restriction, and at 12 months. Samples of serum were obtained by centrifugation at 4000 rpm for 10 min. Samples were stored at −80 °C until analysed. Analysis was performed by ELISAKit.com (Melbourne, Australia).

### 2.4. Ethics

This study was approved by the University of South Australia’s Human Research Ethics Committee (protocol number 0000031828). All participants gave written informed consent. This trial is registered with the Australian New Zealand Clinical Trials Registry (ACTRN12614001041640).

### 2.5. Statistical Analysis

All data is presented as mean ± SD, unless otherwise indicated. A significance level of *p* < 0.05 for each contrast was used. All data were analysed using SPSS (IBM SPSS Statistical Software version 22.0 for Windows, SPSS analysed Inc., Chicago, IL, USA). Graphs and tables were created using Microsoft Excel 2013 for Windows (Microsoft Inc.). A general linear model of repeated measures was used to calculate time, diet and time x diet interactions for the outcome variable. 

## 3. Results

Baseline characteristics of participants included in this analysis are outlined in Table 1. Only BMI was significantly different between the two groups. 

As expected after 12 months there was a reduction in weight and BMI within both groups (Table 2). FGF-21 levels were not altered by weight loss and were not correlated with body weight or BMI at baseline or 12 months. Furthermore, change in FGF-21 was not correlated with change in weight.

## 4. Discussion

In this exploratory study there was no change in FGF-21 concentrations between baseline and 12 months. Human studies assessing the impact of weight loss on FGF-21 levels are limited and have conflicting results. Studies have shown FGF-21 levels decreasing [8] or remaining the same [9] following weight loss after dietary intervention, decreasing after gastric banding [8] and sleeve gastrectomy [10], and increasing [8,11] or no change [12] following weight loss after Roux-en-Y gastric bypass (RYGB). The designs of these studies vary, from number and type of participants, type of intervention, time frame following intervention type and outcome measurement, as well as amount of weight loss. Therefore, our study showing no change in FGF-21 concentrations after 12 months of either CER or IER is not entirely unexpected.

The conflicting results, albeit in a minimal number of studies, of the impact of weight loss following dietary interventions on FGF-21 suggest that FGF-21 levels may not be directly regulated by changes in fat and mass and body weight. Christodoulides et al. [14] showed that an average weight loss of 9.2%, induced by a ketogenic diet, resulted in a 42% difference in FGF-21 levels. Similarly, Gomez-Ambrosi et al. [15] reported a decrease in FGF-21 following weight loss, after diet therapy (−11 ± 5 kg) or surgical intervention (sleeve gastrectomy −27 ± 5 kg and Roux-en-Y gastric bypass −40 ± 14 kg). Conversely, Mai et al. [10] reported that despite weight loss (5.3%), FGF-21 levels remained the same. This suggests that the degree of weight loss in our current trial (6%) may not have been great enough to see any changes in FGF-21. Modest weight loss, as seen in the current trial, is common throughout weight-loss literature as well as clinical practice, with the impact of larger weight loss (i.e., weight loss experienced after surgical intervention) on FGF-21 levels requiring further research.

Most recently, Sanyal et al. [16] assessed the safety and efficacy of Pegbelfermin (an FGF-21 analogue) in patients with non-alcoholic steatohepatitis, a condition commonly associated with overweight/obesity. Findings showed a significant decrease in absolute hepatic fat fraction in the group of patients receiving either 10 mg once a day or 20 mg Pegbelfermin weekly compared to the placebo group, with no associated changes in bodyweight—indicating that increased levels of FGF-21 can assist with a reduction of liver fat, even without significant weight loss.

In the liver, FGF-21 induces fatty acid oxidation, ketogenesis and gluconeogenesis [4,5,17]. Studies in mice have shown that FGF-21 is not involved in glycogenolysis, which occurs in the early stages of fasting, but rather during prolonged fasting, when glycogen stores are depleted [17]. This has also been confirmed in humans, where FGF-21 concentrations were induced only after prolonged fasting [18]. FGF-21 has been linked to hepatic metabolism via, at least in part, PGC-1alpha. The act of fasting induces PGC-1alpha in the liver, which in turn stimulates genes involved in fatty acid oxidation, gluconeogenesis and ketogenesis [19,20,21]. Furthermore, mice lacking FGF-21 have failed to fully induce PGC-1alpha expression in response to a prolonged fast and shown to have reduced ketogenesis and gluconeogenesis [17]. The fact that we did not see a change in FGF-21 levels in the current study suggests that the energy restriction prescribed was potentially not great enough to mimic a fasting state. 

Another factor impacting the ability to see changes in FGF-21 weight loss via dietary interventions maybe the type of diet used. FGF-21 appears to play a role in ketogenesis [22], and therefore it is potentially only dietary interventions designed to induce pronounced ketosis that will be effective in modifying FGF-21. Christodoulides et al. [14] used a ketogenic diet, compared to Mai et al. and our current study that did not, possibly explaining the difference in results found. It is important to note, however, that a trial investigating the impact of a ketogenic diet in children did not report a change in FGF-21 levels [18]. Furthermore, Mraz et al. [23] showed an increase in FGF-21 levels after three weeks of a very low energy diet (resulting in moderate weight loss). This increase in FGF-21 may be explained, though, through the catabolism that occurs after following a very low energy diet, making a direct comparison between this trial and our results difficult. 

This long-term randomised control trial in healthy overweight or obese individuals is adding to the growing literature investigating the role that FGF-21 may have in obesity and associated weight loss. However, the number of participants used for analysis should be considered when considering the generalizability of results. The small number used in this exploratory analysis may have meant that it is not adequately powered to observe any potential changes in FGF-21 levels. The variance between individuals’ response of other hormones involved in energy homeostasis, and how this impacts FGF-21, is also a key area that should be explored. 

## 5. Conclusions

In conclusion, in this exploratory study, weight loss after 12 months either through CER or IER did not result in a change in FGF-21 level in healthy subjects with overweight or obesity. As there was no change in FGF-21 levels from baseline to 12 months in the sub cohort used for analysis, further investigations were not conducted. 

## Figures and Tables

**Table 1:** Baseline characteristics of the participants.

Characteristic	Whole Cohort (*n* = 43)	CER (*n* = 23)	WOWO (*n* = 20)
Age	53.3 ± 9.9	53.7 ± 7.7	52.9 ± 12.2
Sex, *n* (%)			
Male	11 (25.6)	6 (26)	5 (15)
Female	32 (74.4)	17 (74)	15 (75)
Weight, kg	90.1 ± 13.9	86.8 ± 11.7	93.9 ± 15.6
BMI, kg/m^2^	32.3 ± 3.7	31.4 ± 2.9	33.2 ± 4.3*
FGF-21 (ng/mL)	0.8 ± 1.1	1.0 ± 1.4	0.5 ± 0.8

* = significantly different to CER (*p* < 0.05).

**Table 2:** Changes to outcome measurements.

	CER			WOWO			Change		
	Baseline	12 Months	*p*	Baseline	12 Months	*p*	CER	WOWO	*p*
**Weight, kg**	86.8 ± 11.7	80.9 ± 11.6	<0.01	93.9 ± 15.6	89.0 ± 16.0	<0.01	−5.9± 4.5	−4.9± 3.4	0.5
**BMI, kg/m^2^**	31.4 ± 2.9	29.3 ± 3.0	<0.01	33.2 ± 4.3	31.5 ± 4.7	<0.01	−2.1 ± 1.6	−1.7± 1.2	0.4
**FGF-21, ng/mL**	1.0 ± 1.4	1.3 ± 1.9	0.09	0.5 ± 0.8	0.6 ± 0.9	0.24	0.3 ± 0.9	0.04 ± 0.2	0.2

## References

[1] Owen B.M., Mangelsdorf D.J., Kliewer S.A. (2015). Tissue-specifc actions of the metabolic hormones FGF15/19 and FGF21. Trends Endocrinol. Metab..

[2] Angelin B., Larsson T.E., Rudling M. (2012). Circulating fibroblast growth factors as metabolic regulators—A cricitcal apprasial. Cell Metab..

[3] Fisher F.M., Chiu P.C., Antonellis P.J., Bina H.A., Kharitonenkov A., Flier J.S., Maratos-Flier E. (2010). Obesity Is a Fibroblast Growth Factor 21 (FGF21)-Resistant State. Diabetes.

[4] Badman M.K., Pissios P., Kennedy A.R., Koukos G., Flier J.S., Maratos-Flier E. (2007). Hepatic fibroblast growth factor 21 is regulated by PPAR-alpha and is a key mediator of hepatic lipid metabolism in ketotic states. Cell Metab..

[5] Inagaki T., Dutchak P., Zhao G., Ding X., Gautron L., Parameswara V., Li Y., Goetz R., Mohammadi M., Esser V. (2007). Endocrine regulation of the fasting response by PPARalpha-mediated induction of fibroblast growth factor 21. Cell Metab..

[6] Lundasen T., Hunt M.C., Nilsson L.M., Sanyal S., Angelin B., Alexson S.E., Rudling M. (2007). PPAR-alpha is a key regulator of hepatic FGF21. Biochem. Biophys. Res. Commun..

[7] Kliewer S.A., Mangelsdorf D.J. (2010). Fibroblast growth factor 21: From pharmacology to physiology. Am. J. Clin. Nutr..

[8] Lips M.A., de Groot G.H., Berends F.J., Wiezer R., van Wagensveld B.A., Swank D.J., Luijten A., van Dijk K.W., Pijl H., Jansen P.L. (2014). Calorie restriction and Roux-en-Y gastric bypass have opposing effects on circulating FGF21 in morbidly obese subjects. Clin. Endocrinol..

[9] Mai K., Schwarz F., Bobbert T., Andres J., Assmann A., Pfeiffer A.F.H., Spranger J. (2011). Relation between fibroblast growth factor-21, adiposity, metabolism, and weight reduction. Metab. Clin. Exp..

[10] Haluzikova D., Lacinova Z., Kavalkova P., Drapalova J., Krizova J., Bartlova M., Mraz M., Petr T., Vitek L., Kasalicky M. (2013). Laparoscopic sleeve gastrectomy differentially affects serum concentrations of FGF-19 and FGF-21 in morbidly obese subjects. Obesity (Silver Spring).

[11] Jansen P.L., van Werven J., Aarts E., Berends F., Janssen I., Stoker J., Schaap F.G. (2011). Alterations of hormonally active fibrobalst growth factors after Roux-en Y gastric bypass surgery. Dig. Dis..

[12] Woelnerhanssen B., Peterli R., Steinert R.E., Peters T., Borbely Y., Beglinger C. (2011). Effects of postbariatic surgery weight loss on adipokines and metabolic parameters: Comparison of laparoscopic Roux-en Y gastric bypass and laparoscopic sleeve gastrectomy—A prospectice randomized trial. Surg. Obes. Relat. Dis..

[13] Headland M.L., Clifton P.M., Keogh J.B. (2019). Effect of intermittent compared to continuous energy restriction on weight loss and weight maintenance after 12 months in healthy overweight or obese adults. Int. J. Obes..

[14] Christodoulides C., Dyson P., Sprecher D., Tsintzas K., Karpe F. (2009). Circulating FGF21 is induced by PRAR agonists but not ketosis in man. J. Clin. Endocrinol. Metab..

[15] Gomez-Ambrosi J., Gallego-Escuredo J.M., Catalan V., Rodriguez A., Domingo P., Moncada R., Valenti V., Salvador J., Giralt M., Villarroya F. (2017). FGF19 and FGF21 serum concentrations in human obesity and type 2 diabetes behave differently after diet- or surgically-induced weight loss. Clin. Nutr..

[16] Sanyal A., Charles E.D., Neuschwander-Tetri B.A., Loomba R., Harrison S.A., Abdelmalek M.F., Lawitz E.J., Halegoua-DeMarzio D., Kundu S., Noviello S. (2018). Pegbelfermin (BMS-986036), a PEGylated fibroblast growth factor 21 analogue, in patients with non-alcoholic steatohepatitis: A randomised, double-blind, placebo-controlled, phase 2a trial. Lancet.

[17] Potthoff M.J., Inagaki T., Satapati S., Ding X., He T., Goetz R., Mohammadi M., Finck B.N., Mangelsdorf D.J., Kliewer S.A. (2009). FGF21 induces PGC-1alpha and regulates carbohydrate and fatty acid metabolism during the adaptive starvation response. Proc. Natl. Acad. Sci. USA.

[18] Galman C., Lundasen T., Kharitonenkov A., Bina H.A., Eriksson M., Hafstrom I., Dahlin M., Amark P., Angelin B., Rudling M. (2008). The circulating metabolic regulator FGF-21 is induced by prolonged fasting and PPARalpha activation in man. Cell Metab..

[19] Burgess S.C., Leone T.C., Wende A.R., Croce M.A., Chen Z., Sherry A.D., Malloy C.R., Finck B.N. (2006). Diminished hepatic gluconeogenesis via defects in tricarboxylic acid cycle flux in peroxisome proliferator-activated receptor gamma coactivator-1alpha (PGC-1alpha) deficient mice. J. Biol. Chem..

[20] Yoon J.C., Puigserver P., Chen G., Donovan J., Wu Z., Rhee J., Adelmant G., Staffor J., Kahn C.R., Grannfer D.K. (2001). Control of hepatic gluconeogenesis through the transcriptional coactivator PGC-1. Nature.

[21] Rhee J., Inoue Y., Yoon J.C., Puigserver P., Fan M., Gonzalez F.J., Spiegelman B.M. (2003). Regulation of hepatic fasting response by PPARgamma conactivator 1 alpha (PGC-1): Requirement for hepatocyte nuclear factor 4alpha in gluconeogenesis. Proc. Natl. Acad. Sci. USA.

[22] Kharitonenkov A., Shanafelt A.B. (2008). Fibroblast growth factor-21 as a therapeutic agent for metabolic diseases. BioDrugs.

[23] Mraz M., Bartlova M., Lacinova Z., Michalsky D., Kasalicky M., Haluzikova D., Matoulek M., Dostalova I., Humenanska V., Haluzik M. (2009). Serum concentrations and tissue expression of a novel endocrine regulator fibroblast growth factor-21 in patients with type 2 diabetes and obesity. Clin. Endocrinol..

